# Fundus Photography as the Base of Three-Tier Diabetic Retinopathy Care System to Its Wider Roles: Learning from a Case Experience

**DOI:** 10.31729/jnma.5565

**Published:** 2021-01-31

**Authors:** Madhur Dev Bhattarai, Raba Thapa, Manil R Bajracharya, Lochan Karki, Budda B Karki, Alark D Rajouria

**Affiliations:** 1 Ex-unit chief Bir Hospital, Nepal Diabetes Association, Kathmandu, Nepal; 2 Tilganga Institute of Ophthalmology, Kathmandu, Nepal; 3 Bir Hospital, National Academy of Medical Sciences, Kathmandu, Nepal

**Keywords:** *diabetes*, *diabetic retinopathy*, *fundus photography*, *NCD*, *WHO PEN*

## Abstract

A case experience of initiating the ocular fundus photography (OFP) service in a diabetes outpatient clinic of a tertiary-care institution is presented. In the community and within the hospitals, the OFP helps to develop the three-tier diabetic retinopathy (DR) care system comprising: OFP-based DR screening and monitoring, an experienced ophthalmologist in laser therapy, and vitreo-retina specialist services. After three to six months of training, non-ophthalmic allied health professionals could also grade the DR. We also learned that such training program, however, requires broadening to encompass diabetes and major non-communicable diseases comprehensively to fulfill the need of the primary care nurses in health care settings and the full-time job and professional career for them. Medical students and residents now need to be ‘directly’ trained in the interpretation of OFP. The stakeholders involved in public health and medical education may recommend to the public hospitals and medical colleges for the provision of OFP service.

## INTRODUCTION

Diabetes is one of the fastest-growing global health emergencies of the 21st century.^1,2^ Almost 80% of people with diabetes live in low and middle-income countries and are the working-age group.^1,3,4^ Vision-threatening diabetic retinopathy (VTDR) can be present even in newly diagnosed diabetic patients.^5^ There are cost-effective treatments for VTDR.^6^ However, awareness about diabetic retinopathy (DR) may be low.^7^ A case experience of initiation of ocular fundus photography service to regularly screen DR in diabetes outpatient clinic in a tertiary care institution is presented. The five learning points from the case experience are discussed with their practical implications.

## CASE EXPERIENCE OF OCULAR FUNDUS PHOTOGRAPHY (OFP)

**Figure 1:**
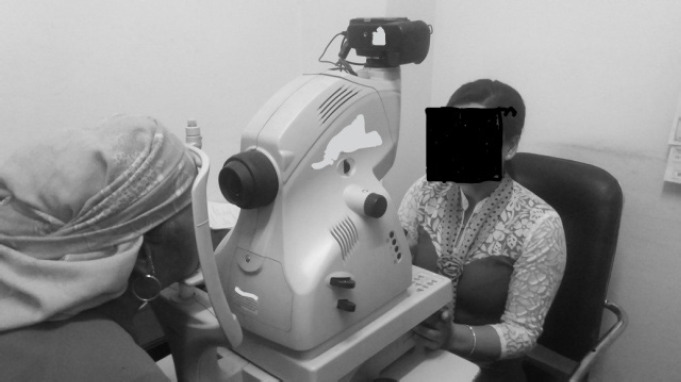
A patient with diabetes undergoing ocular fundus photography (OFP) at the site in diabetes and endocrinology outpatient clinic of Bir Hospital for screening and monitoring of diabetic retinopathy (DR).

In the process of initiating the sub-specialist program of a doctorate in medicine in diabetes and endocrinology about a decade back, the service in the specialty in the tertiary care government institution Bir Hospital, National Academy of Medicine (NAMS), Kathmandu was strengthened. With the six days a week diabetes and endocrinology general outpatient clinic, the flow of the diabetic patients increased. However, there was difficulty getting the routine eye examination of the patients for diabetic retinopathy (DR) regularly. The patients had to be referred to the eye outpatient clinics. The patients needed to visit the eye clinics, and if dilatation of the pupils was to be done, it required extra time or visit and even a company to travel. We noticed that people with diabetes often tended to attend the eye centers only when they developed some vision problem. To make it easier for the diabetic patients to get their regular eye examination for DR done on the spot in the same visit, the need for an ocular fundus camera in diabetes and endocrinology outdoor was felt. An understanding was made with the DR project, supported by the Fred Hollows Foundation, of the Tilganga Institute of Ophthalmology in the year 2013 for the OFP service by the ophthalmic assistants in diabetes and endocrinology outdoor clinic. Since then, it has become easy to regularly perform on-the-spot DR screening of the patients attending the diabetes outpatient clinic from different parts of the country (Figure 1) and to timely refer to the eye clinics for its management before it severely affects the vision.

To run the OFP service on the long-term basis and for its sustainability, it was felt that the local health staff needed to manage it. The DR project, supported by the Fred Hollows Foundation and World Diabetes Foundation of the Tilganga Institute of Ophthalmology, conducted healthcare professionals’ training with certificate level of education in nursing and health with the involvement of diabetes and endocrinology unit. The training was conducted for seven hours each day for six months covering diabetes, OFP, and DR grading and management as well as history taking, visual acuity assessment, and detailed anterior segment evaluation of the eye using torchlight.^8^ The training report revealed that the non-ophthalmic allied health professionals could perform OFP and grade DR and refer to the ophthalmologists. It was also noted that the training could be reduced to even three months duration.^8^

The Souvenir annual report published on the occasion of the 131^st^ anniversary of Bir Hospital reported that more than 23 thousand new and follow-up patients with diabetes, thyroid, and other endocrinology disorders attended diabetes and endocrinology outpatient clinic last year. It was the highest number among the patients attending various medical sub-specialty outpatient clinics in the hospital. Similarly, the number of ocular fundus photography (OFP) done last year in the outpatient clinic was about 12 hundred. Apart from utilizing the OFP for DR screening and education of the diabetic patients and the training of the health care professionals in OFP, it was also beneficial to the patients with various other systemic diseases having retinal manifestations. The OFP was equally useful for the learning of the non-ophthalmologist residents and fellows in the manifestations of various systemic health conditions in the retina.

## OCULAR FUNDUS PHOTOGRAPHY (OFP) AT THE SITE IN THE OUTPATIENT CLINICS OF DIABETES MANAGEMENT

The experience of initiating the OFP service in diabetes and endocrine outpatient clinic in the tertiary care institution provided us several insights. From our experience, we learned that if there are no visual symptoms, people with diabetes may not be inclined to make extra visits to the eye clinics for routine diabetic retinopathy (DR) screening and monitoring. On-the-spot availability of the OFP in the outpatient clinic of diabetes encourages people with diabetes with or without vision impairment to get their retina examined regularly. The current opportunistic DR method using mydriatic bio-microscopic ophthalmoscopy by an ophthalmologist in most low and middle-income countries is not an efficient way of screening for DR considering the limitations in human resources and access barriers.^9^ Much training and skill of health care workers is often required for indirect ophthalmoscopy and slit-lamp biomicroscopy.^3^ As such, in Nepal, only limited public hospitals have separate eye departments.^8^ OFP is increasingly used for diabetic retinopathy (DR) screening globally, including in industrialized countries.^6,9^ Non-mydriatic two-field strategy is a pragmatic approach in initiating DR screening programs for people with diabetes in low-income settings and dilatation of the pupils of those who have ungradable images.^9^ Non-mydriatic retina imaging is now recommended for screening of people with diabetes in all non-ophthalmic settings.^3,6,9^

## OCULAR FUNDUS PHOTOGRAPHY (OFP) BY NON-OPHTHALMIC HEALTH PERSONNEL

Next, we learned that the non-ophthalmic allied health professionals could provide the service of OFP after three to six months of training with adequate exposure to retinal imaging.^8^ The prevalence of diabetes is more in urban than in rural areas; however, it is increasing in both areas globally.^2^ At the beginning of the diabetes epidemic, diabetes was most prevalent in Nepal's urban community, with the initial indication of its rising trend in the hospitals.^10,11^ Diabetes is now getting prevalent all over the country, more in the urban areas. A study reported up to 7.9% prevalence of gestational diabetes in a rural community in Nepal.^12^ Such an increasing prevalence of diabetes in the rural areas could be due to the changing lifestyle and migration of people and the availability of remittance sent by family members working abroad. The rural people going abroad for work are also at a similar risk of developing diabetes and its complications like cardiovascular disease.

The age-adjusted comparative prevalence of diabetes in adults 20-79 years in Nepal is 7.2%, with about 700 thousand people with diabetes in the country.^1^ It will not be immediately possible to provide DR screening service all over the country by establishing eye centers with ophthalmic medical and health professionals. Diabetic retinopathy (DR) screening by ophthalmologists is not an efficient way of screening for any setting.^9^ After the training in OFP, the much larger pool of workforce of non-ophthalmic personnel, can perform DR screening using digital imaging.^6,9^ It is now recommended to train non-ophthalmic personnel in DR grading, just as it is done in different national programs, including in the industrialized countries.^3,6,9^

## THREE-TIER DIABETIC RETINOPATHY (DR) CARE SYSTEM IN THE COMMUNITY

Thirdly, we learned that the OFP service available as the base for regular eye screening at the site of assessment of diabetes helps to develop the three-tier DR care system for people with diabetes. Overall, about one-third of the people with diabetes develop DR, and about one-third of those with DR develop vision-threatening DR (VTDR).^1,4,9^ With such proportions out of the estimated 700 thousand people with diabetes,^1^ the number of people with DR will be about 250 thousand and with VTDR about 80 thousand in Nepal. However, the prevalence of DR in people with diabetes is reported to be lower in the South Asian populations and other developing countries.^5,6^ Such lower DR prevalence may be due to a more recent increase in the prevalence of diabetes in Asian populations and the increased age of the diabetic population, and a longer life expectancy in the industrialized world.^5,6^ To estimate the number of people with DR in India, it was assumed that 15-20% of the people with diabetes have any DR, and 5-7% VTDR.^6^

**Figure 2:**
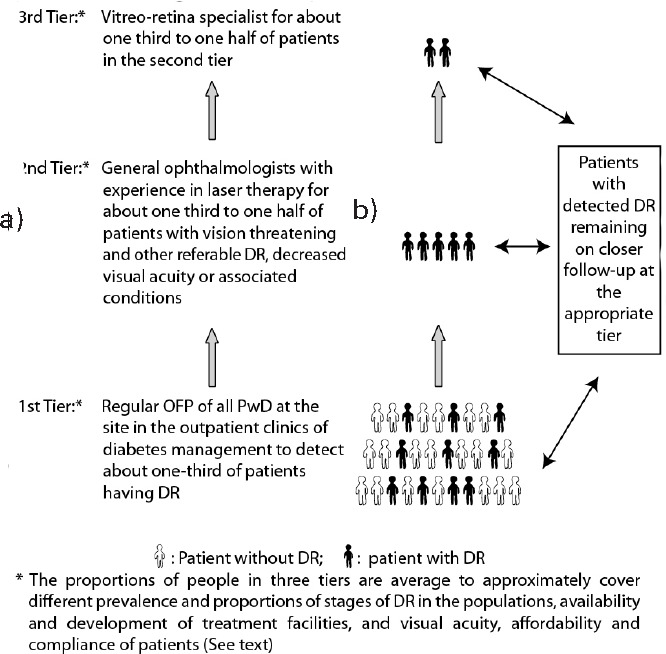
Diagrammatic representation of the proportions of people in the three-tier diabetic retinopathy (DR) care system with the ocular fundus photography (OFP) at the site in the outpatient clinics of diabetes management as the base to screen all people with diabetes (PwD).

Local and regional guidelines of DR are increasingly available. As per the stages of DR and compliance of the patients for follow-up and their affordability, different available treatments, singly or in combination, can be used like laser pan-retinal photocoagulation (PRP), focal/grid laser, intravitreal anti-vascular endothelial growth factor (anti-VEGF) and steroid injections, and vitreoretinal surgery.^3,6^ Accordingly, with the OFP service as the base for regular DR screening, most people with diabetes may not be required to be referred to ophthalmologists. In this way, the OFP helps develop the three-tier DR care system comprising: OFP-based DR screening and monitoring, experienced ophthalmologist in laser therapy, and vitreo-retina specialist services (Figure 2). Depending on their preexisting experience, general ophthalmologists can undergo short-term laser therapy training as per their requirements. Such modular training is conducted in Nepal by the Tilganga Institute of Ophthalmology.

The three-tier diabetic retinopathy (DR) care system (Figure 2) in the community appears to have other beneficial effects also. It tends to decrease the unnecessary visit of the bulk of the people with diabetes, not requiring any interventions for regular screening of DR to the ophthalmologists and tertiary care centers. This will help the patients requiring further treatment to get an appointment in the eye centers for the appropriate management without any delay. The eye centers and ophthalmologists can also better focus on the patients requiring expert care. With timely screening and management of patients with DR, increasing experience, and availability of human resources, technologies, and medications, the proportion of patients requiring referral to the tertiary care levels will further decrease.

## THREE-TIER DIABETIC RETINOPATHY (DR) CARE SYSTEM WITHIN THE HOSPITALS

If the service of the OFP is initiated in a general hospital or medical college, the institution can itself also get benefitted from the increased visit of the people with diabetes and by the referral from other physicians. The institution can further develop laser treatment and other technology, like fundus fluorescein angiography, ocular ultrasound, and optical coherence tomography (OCT), for DR management, attracting the ophthalmologists to join the institution. Thus, the OFP service initiation can create a snowball effect of expanding the hospital's service and human resources, and the three-tier DR care system can be developed within the institution itself.

## COMPREHENSIVE EDUCATION OF DIABETIC PATIENTS AND PROFESSIONAL CAREER OF THE HEALTH PERSONNEL

Fourthly, we learned that the process of undergoing OFP and visualizing the retinal imaging help the diabetic patients to be aware of the need for regular DR screening and management of its risk factors like chronic hyperglycemia, hypertension, nephropathy, dyslipidemia, and pregnancy. However, we noticed that though the health professionals performing the OFP tried their best to educate diabetic patients about DR management and control of its risk factors, they could not do so comprehensively about diabetes management. Further training in comprehensive diabetes management can increase their utility in this situation greatly to the patients. The patients require comprehensive educational information and follow-up sessions by competent professionals to self-manage safely. The physicians often have only time to address the immediate problem during a patient visit.^13,14^ Similarly, the other related issue, we realized, is the need for full-time job allocation and professional career for the health care professionals performing the OFP only. The training in the OFP requires broadening to encompass diabetes management comprehensively to fulfill the need not only of the primary care of the patients but also of the full-time job and professional career for the trainees.

## COMPREHENSIVE DIABETES AND NON-COMMUNICABLE DISEASE (NCD) EDUCATOR

In industrialized countries, the health care professionals like nurses are trained as Diabetes Educator to provide comprehensive primary care service and education to diabetic patients in the hospitals and general practice under the supervision of medical professionals.^13,14^ In developing countries like Nepal, the practice of diabetes educator is still uncommon.^14^ With the WHO Package of Essential NCD (WHO PEN) Interventions for primary care being increasingly implemented in the districts,^15^ there is now a need of the training of primary care health professionals for major NCD to provide necessary primary care and education to the patients under the supervision of the health unit in-charges.^14^ The four major NCD common in the population all over the world include diabetes, cardiovascular diseases (CVD), chronic respiratory diseases, and cancer. They share common risk factors, like physical inactivity, unhealthy diet, obesity, smoking, alcohol misuse, hypertension, and dyslipidemia.^15^ Further, as they all are common and share common risk factors, the four major NCD often coexist. Moreover, CVD is a common complication causing morbidity and mortality in diabetes. It is just recently reported that the CVD rates, CVD mortality, and all-cause mortality are markedly higher among those with diabetes in low-income countries than others.^16^ Apart from controlling and monitoring blood glucose, the management of diabetes includes the management of its risk factors, including obesity and comorbidities, especially hypertension, and the prevention of the complication of CVD.^17^ The inclusion of the use of inhaled steroids and bronchodilators in chronic respiratory disease and identification of presenting features of cancer along with other interventions indicated in the WHO PEN in the curriculum can complete the areas of training of traditional diabetes educator as that of educator for the four major NCD for primary care and education of the patients.^14,15^

## STAFF NURSE TRAINING AS THE MAJOR NCD PRIMARY CARE ASSISTANT

One year Major NCD Primary Care Assistant training curriculum for staff nurses with certificate level of education in nursing has recently been designed by the collaboration of the experts from different fields viz. health ministry and department; Apex Body of Eye Health; curriculum division; hospitals, including Bir Hospital; associations, like Nepal Diabetes Association; and nursing, gynecology, ophthalmic and different medical specialties. The DR project facilitated the curriculum development, supported by The Fred Hollows Foundation and World Diabetes Foundation, of the Tilganga Institute of Ophthalmology. The training program is required to be structured, covering the essential and cost-effective affordable technology and interventions of the WHO PEN for diabetes and other major NCD that are feasible and planned for implementation in primary care in low resource settings.^14,15^ Table 1 shows the outline of the designed training program of the major non-communicable disease primary care assistant.

**Table 1:** Outline of the training program of the Major Non-Communicable Disease (NCD) Primary Care Assistant

Eligibility for enrollment	Staff nurse of certificate level of education in nursing with a minimum of two years practice experience
Duration of the course	One year with about two-thousand hours of hands-on working experience
Postings in different specialties on rotation for hands-on working experience	Adult and pediatric diabetes (including fundus photography), cardiology, pulmonary, neurology (for stroke), nephrology (for diabetic nephropathy and renal replacement therapy), oncology (including prevention and early detection of cancers and palliative care), ophthalmology, and obstetrics and gynecology (including gestational diabetes, promotion of breast feeding, and early detection of cancers in women) units
Academic sessions	Lecture, presentation and discussions of two hundred hours as per the curriculum topics covering four major NCD Case-based discussions of the experience in different specialty units Discussion on the counseling experience with the patients done with or without the help of handy flip charts as the printed folders or in the electronic form in the Tablet in the diabetic and other units Mandatory training to cover counseling process, inhaler therapy, breastfeeding counseling, glycemic management to educate self-management to the patients, and others
Case history recording	About two hundred fifty case history recordings while posted in different specialty units
Logbook	Documentation of the academic and posting activities and the procedures performed as per the minimum number specified for each trainee
Assessment	Formative and summative theory and clinical practical examinations

Staff nurses regularly work under medical doctors’ supervision in hospitals and in-charges of primary care health facilities. Their working as Major NCD Primary Care Assistants from primary to tertiary care health facilities would be in accordance with the health system's prevailing routine functioning and hierarchy. The staff nurses with one year of Major NCD Primary Care Assistant training would be quite appropriate for the optimum implementation of the districts’ WHO PEN interventions. The training of staff nurses as Major NCD Primary Care Assistant can, thus, consolidate the care and preventive service of diabetic retinopathy and diabetes and other major NCD and provide the trained personnel a full-time job and a professional career.

## OCULAR FUNDUS PHOTOGRAPHY (OFP) IN CLINICAL AND RESEARCH FIELDS

Finally, we learned that documentation of retinal imaging by the OFP was also useful to the patients with various other diseases having retinal manifestations. It was equally beneficial for the non-ophthalmology residents and fellows in various manifestations of systemic health conditions in the retina. Apart from the DR, the OFP is useful to detect various retinal findings, like hypertensive retinopathy, retinal vascular occlusion, papilloedema, optic disc pallor, hemorrhage, exudates, retinoblastoma, retinopathy of prematurity, and others.^18–22^ Many of such retinal findings are due to the diseases of various organs and body systems other than the eyes. Advantages of OFP are many, including its ability to make the permanent record of a patient's ocular fundus appearance for objective analysis, to electronically magnify regions of interest in photographs, to transfer the imaging for remote interpretation, to analyze the changes in the retinal vasculature by a computer program, and to use it as an educational tool for the patients and trainees.^3,6,18–21^ The fundus photographs can be printed and stored in electronic form. Serial imaging is very useful in detecting changes over time and assessing the response to treatment.^6^ Embryologically, the retina is an extension of the diencephalon of the brain.^18^ Retinal vasculature is the mirror of that of the brain. The OFP makes it possible for the physicians to visualize the vasculature for cardiovascular disease education, prevention, management, and research. Being easily accessible and predominantly automated and objective, retinal imaging has emerged as an exciting research tool for cardiovascular risk assessment.^18,20^

Ocular fundus photography (OFP) directly linking the non-ophthalmologists with retinal imaging: Visualizing the ocular fundus is essential in medicine. There are many systemic conditions, other than the local eye diseases, which can affect the retina. Various ophthalmic manifestations have been listed under the headings of major body systems and conditions in different tables in the standard textbook of medicine for medical residents and students under a separate new chapter of medical ophthalmology.^22^ The lists and the approaches are likely to be more elaborated in the future. The chapter is aptly named the medical ophthalmology, not as eye diseases or disorders, as there are many ophthalmic conditions where systemic diseases and drugs have to be considered and managed.^22^ Even in the ophthalmic conditions like refractive errors, age-related cataract, and macular degeneration, glaucoma, congenital anomalies, other degenerative conditions, and local infections, tumor, and trauma, the systemic conditions and drugs may also need to be considered or managed. Direct ophthalmoscopy has been the traditional method of visualizing the retina. However, it requires adequate training and practice and is infrequently and poorly performed by most non-ophthalmologist physicians, including neurologists, due to the technical difficulty related to direct ophthalmoscopy.^19^ Pharmacologic dilation of the pupil is routinely utilized by ophthalmologists in their examination of the ocular fundus; however, it is almost never performed in non-ophthalmic settings, largely due to lack of access to dilating drops, fear of side effects, and reluctance to wait for adequate pupillary dilation.^19^ For non-ophthalmology students, residents, fellows, and faculty, the OFP has now made available the retinal imaging in printed and electronic forms for objective observation, interpretation, and discussion like the electrocardiogram (ECG) and chest X-ray. Studies have documented that nonmydriatic OFP is an effective alternative way of providing access to the ocular fundus in non-ophthalmic settings like the emergency department.^19^

The OFP has indeed fulfilled the dire need for a method for non-ophthalmologists to visualize the retina. Recent advances in portable easy-to-operate inexpensive fundus cameras and smartphone-based fundus imaging systems have revolutionized retinal imaging, which has grown exponentially over the last decade.^21^ In the future, with artificial intelligence, the machine-generated reports, including the arteriovenous ratio, can further help interpret retinal imaging. The non-ophthalmic allied health care professionals like nurses are grading the DR routinely by OFP. The medical students and residents now need to be ‘directly’ trained in interpreting fundus photographs.

**Table 2:** The five learning points from the experience of initiating the ocular fundus photography (OFP) service on-the-spot in diabetes and endocrine outpatient clinic.

If there is no visual symptom, the people with diabetes may not be inclined to make extra visits to the eye clinics for routine diabetic retinopathy (DR) screening. With on-the-spot OFP service available in the outpatient clinic of diabetes management, DR screening of the patients can be done regularly. The non-ophthalmic health personnel can provide the OFP service after three to six months of training. The OFP helps to develop in the community and within the hospitals, the three-tier DR care system comprising: OFP-based DR screening and monitoring, experienced ophthalmologist in laser therapy, and vitreo-retina specialist services. The experience of and interaction for undergoing OFP and visualizing the retinal imaging makes the diabetic patients aware of DR and its risk factors. However, they require much educational information. The training of health personnel in OFP requires broadening to encompass diabetes and major non-communicable diseases comprehensively to fulfill the need not only of the full-time job and professional career for the trainees but also of the primary care nurses in health care settings for the patients, as the physicians are often busy addressing the immediate problems of the patients. The OFP is also beneficial in managing many systemic diseases, apart from diabetes, having retinal manifestations. It is equally useful for the learning of the non-ophthalmologist residents and fellows. The OFP has now 'directly' linked the non-ophthalmologist physicians with retinal imaging for patient management, public health, medical education, and research.

## THE WAY FORWARD

The five learning points from the experience of initiating the ocular fundus photography (OFP) on-the-spot in diabetes and endocrine outpatient clinic are summarized in Table 2. The OFP complements the highly effective treatment available for the prevention of blindness in people with diabetes. It effectively links the mass of the diabetic patients with the class of ophthalmic service and the non-ophthalmic medical professionals ‘directly’ with the retinal imaging. The OFP service can be the base for developing a a three-tier diabetic retinopathy care system in any institution or community. Both public and private medical institutions can effectively utilize it for the care and education of patients with diabetes and other diseases; for the training of medical students and residents in various retinal findings; and for the research incorporating retinal features of diabetes, hypertension, atherosclerosis, and various other systemic conditions. The portable OFP can be used in the health camps in the community to care for and educate people with diabetes. Interpretation of fundus photographs has become essential in clinical management, medical education, research, and public health. Commissions, councils, and universities supervising the medical education may recommend teaching medical institutions to provide the OFP service for the benefit of patients, medical students, residents, and other trainees. Units of the ministry and department of health and public health organizations involved in diabetes and eye care may prioritize making the OFP service available in the community to prevent blindness in the population.
